# Commentary: Determination of the 95% effective dose of remimazolam tosylate in anesthesia induction inhibits endotracheal intubation response in senile patients

**DOI:** 10.3389/fphar.2025.1555758

**Published:** 2025-09-02

**Authors:** Peipei Sun, Tingting Ma, Chen Zhou, Jie Zhou, Bingwei Hu

**Affiliations:** ^1^ Department of Hemodialysis Center, Hangzhou Hospital of Traditional Chinese Medicine, The Affiliated Hospital to Zhejiang Chinese Medicine University, Hangzhou, China; ^2^ Department of Anesthesiology, Tongde Hospital of Zhejiang Province, Hangzhou, Zhejiang, China

**Keywords:** 95% effective dose, biased-coin design, isotonic regression, remimazolam, anesthesia

## Introduction

Medication use in special populations—such as older adults, children, frail patients, and obese individuals—often presents clinical challenges due to insufficient evidence-based guidance. Consequently, research targeting these groups holds significant value for informing pharmacotherapeutic decisions. This imperative extends to emergency surgical patients, whose distinct physiological responses may yield clinically actionable evidence not generalizable from routine populations (Pouraghaei et al., 2013). A review revealed that preoperative frailty is significantly correlated with adverse perioperative outcomes (Kikuchi et al., 2021). Consequently, it is highly important for anesthesiologists to pay more attention to frail patients. In frail patients undergoing general anesthesia, the dose of an anesthetic drug necessary to suppress the response to tracheal intubation might be lower than that in non-frail patients. Therefore, applying the recommended dose for non-frail patients to frail patients might lead to harm to patients, such as hemodynamic fluctuations. Remimazolam, a novel drug, is recommended for anesthesia induction because of its lesser impact on circulation (Hu et al., 2022). Studies have demonstrated that the 50% effective dose (ED50) and 95% effective dose (ED95) of remimazolam for sedation during gastroscopy in elderly patients are 0.153 mg/kg and 0.162 mg/kg (Sun et al., 2022) respectively. This could offer a dose recommendation for remimazolam to suppress the response to tracheal intubation in non-frail elderly patients. However, the ED95 obtained through probit regression based on the ED50 is not reliable, and researchers have proposed biased coin design (BCD).

Qu et al. used a BCD and isotonic regression to determine that the 95% effective dose of remimazolam used for anesthesia induction inhibits the endotracheal intubation response in senile patients (Qu et al., 2023). This is an interesting study, and the results of the precise ED95 are sufficiently attractive for clinical anesthesiologists. Nevertheless, we observe that the results presented in the article are perplexing and that the analysis of the data is incomplete.

### The data of adjusted response rates were not reproducible

Qu et al. assert that isotonic regression is employed to calculate ED95, and the pool-adjacent-violators algorithm (PAVA) is employed to adjust response rates. PAVA replaces the data of adjacent violations with a weighted mean to guarantee the nondecreasing nature of the data, which can be acquired by utilizing the *gpava* function within the isotone package. However, the adjusted response rates of non-fail patients were not in accordance with the PAVA results (Table 1). Particularly in the Frail group, the response rates and doses were monotonically increasing. Thus, the results of PAVA could not alter the data. In reality, simple rules based on PAVA do not have to utilize a computer to obtain an answer. For the aforementioned reasons, the outcomes calculated by authors are highly astonishing.

**TABLE 1:** Adjusted response rates of different algorithms.

Dose	Observed reponse rates	Calculated by Qu et al.	gpava^a^	cirPAVA^b^
Non-frail Group				
0.30	0.929	0.870	0.828	0.828
0.32	0.727	0.920	0.828	0.885
0.34	1	1	1	1
Frail Group				
0.26	0.667	0.630	0.667	0.667
0.28	0.875	0.940	0.875	0.875
0.30	1	1	1	1

Note: ^a^ gpava is a function in the R package of ‘isotone’.

^b^ cirPAVA, is a function in the R package of ‘cir’.

### Point and interval estimations demand further assessment

A linear interpolation was employed to estimate the dose with a target probability of Γ = 0.95, which essentially constitutes a straight line fit with explicit formulas for calculation (Pace et al., 2007). Hence, the ED95 calculated by the Qu et al. on the basis of dubious PAVA-adjusted response rates is also subject to skepticism. The authors assert that the 95% confidence interval (CI) for ED95 was calculated via a *bias-corrected percentile method* (Stylianou M et al., 2003). Software has been written in GAUSS and R (Pace et al., 2007), but the code seems not to be publicly accessible, and researchers have to program it on their own. Discrepancies in code may result in marginally different outcomes. In this algorithm, the interval estimate of the dose is computed on the basis of the inverse function of the empirical cumulative distribution; therefore, the range does not exceed that of the trial doses (Table 2). An R package (https://rdrr.io/cran/ed50/) for ED50 provides a similar calculation, and it is also explicitly indicated that the CIs are bounded as the bootstrap parametric resample function generates additional doses strictly within the dose sample space. We observe that the results of Qu et al. extend far beyond the range of trial doses, a situation that does not seem to align with the *bias-corrected percentile method*. An algorithm known as *centered isotonic regression* improves the PAVA by achieving the monotonically increasing of the adjusted response rates and doses (Oron and Nancy, 2017). The CI with this algorithm would lie outside the range of the trial dose, as it offers an algorithm distinct from the *bias-corrected percentile method*. Consequently, the authors are obligated to verify the correctness of the code.

**TABLE 2:** Calculation results of ED95 and 95% CI for different codes.

Group	Range of dose	ED95 and 95%CI		
		Calculated by Qu et al.	Calculated by us*	Calculated with cir
Non-frail	0.30–0.34	0.331 (0.272–0.472)	0.334 (0.294–0.340)	0.331 (0.318–0.456)
Frail	0.26–0.30	0.297 (0.231–0.451)	0292 (0.263–0.300)	0.292 (0.287–0.406)

Note: * The codes are shown in Supplementary Appendix I.

### Detailed reporting is needed for the statistical analyses of secondary clinical outcomes

Qu et al. reported that DBP was significantly greater in the non-frail group than in the frail group at 1 and 2 min postintubation (p = 0.006 and 0.010, respectively). As depicted in their statistical analysis, two-factor repeated measures analysis of variance (ANOVA) was used to compare the differences between the two groups. The authors fail to report whether the assumptions employed for repeated-measures ANOVA are valid, such as normality and homogeneity of variance across groups and levels. More crucially, the authors fail to report relevant statistics or effect sizes. If normality and homogeneity of variance are not fulfilled, it is suggested that more robust statistical methods, such as generalized estimating equations, be employed. In addition, from Figure 4B in the paper, the baseline heart rate (HR) of the two groups was significantly different (approximately 15 bpm), and it is not evident whether the authors corrected it as a covariate.

**FIGURE 1:**
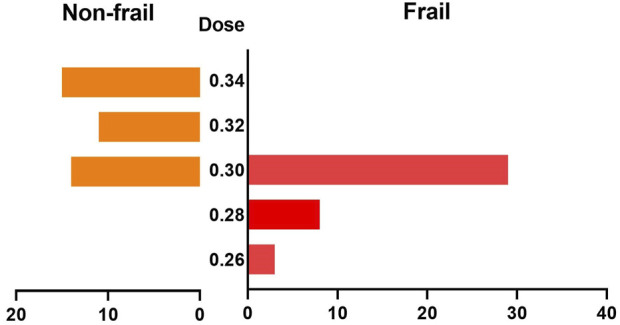
Comparison of the dose distributions between the two groups.

Qu et al. compared the demographic characteristics of the Non-frail group and the frail group to demonstrate the balance of the baseline data of the two cohorts. This is indispensable for the comparison of ED95 between the two cohorts. However, it appears that the authors intend to explore the influences of remimazolam on BP and HR in both cohorts. There is no evidence suggesting that the drug dose has no effect on BP or HR; rather, it is more probable that the drug dose is a confounding factor influencing BP and HR in different cohorts. The proportions of drug doses utilized in the two cohorts were dissimilar (Figure 1), so we propose that the dose should also be incorporated as a covariate in the analysis to clarify whether the variations in BP and HR are attributed to exposure factors (frail) or confounding factors (drug). Given that the authors failed to report detailed information regarding the statistical analyses, the reliability of their results cannot be trusted.

## Conclusion

Dose finding studies typically utilize standardized methodological frameworks and often generate expected results—characteristics that could foster excessive confidence in their conclusions. Although computational discrepancies are relatively rare in dosing trials, they remain a potential concern. We emphasize the importance of critical appraisal when interpreting research outcomes; clinical application of conclusions based on biased evidence poses substantial risks to patient safety. Prior to translating such evidence into practice, thorough validation of the reliability of study conclusions is essential, particularly for findings lacking comprehensive evaluation.

The study conducted by Qu et al., which employed BCD to determine the ED95 of remimazolam in senile patients, is of interest. Nevertheless, the incomplete reporting of data processing has led to doubts regarding the accuracy of the results. As readers, we look forward to more detailed data reports or errata from the authors.
