# Supplementary material for: Commentary: Determination of the 95% effective dose of remimazolam tosylate in anesthesia induction inhibits endotracheal intubation response in senile patients
Source: Front Pharmacol. 2025 Sep 2;16:1555758. doi: 10.3389/fphar.2025.1555758 (PMC12436137; doi:10.3389/fphar.2025.1555758)
Supplement: Supplementary file 1 [file DataSheet2.docx]

# Appendix Ⅱ A Brief Introduction to Statistical Methods

# Probit regression

Probit regression is a statistical method used to model the relationship between one or more predictor variables and a binary outcome (e.g., success/failure). In dose-finding studies, the model is employed to fit the cumulative distribution function of drug response. This allows for the estimation of doses corresponding to specific response probabilities, such as ED₅₀ and ED₉₀. It is important to note that this model rely on the key assumption that the probability density function follows a normal distribution.

# Biased coin design

Biased coin design is a response-adaptive randomization technique used primarily in clinical trials to balance treatment group sizes while incorporating accumulating outcome data to preferentially assign patients to the seemingly superior treatment. In dose-finding studies targeting metrics such as ED90, biased coin design concentrates experimental doses near the target probability threshold (e.g., 0.9), thereby enhancing the precision of parameter estimation.

# Isotonic regression

Isotonic regression is a nonparametric statistical technique that fits a monotonic function to observed data while minimizing the sum of squared errors. Unlike parametric models (e.g., probit regression), it imposes no predefined functional form but strictly enforces that the estimated response function is either entirely non-decreasing or non-increasing. In dose-finding studies, isotonic regression corrects non-monotonic dose-response patterns caused by random variability, ensuring response rates adhere to biological plausibility. The adjusted response rates are subsequently used to fit the empirical cumulative distribution function.

# Pool-adjacent-violators algorithm

Pool-adjacent-violators algorithm is the fundamental computational procedure for solving the isotonic regression problem. It efficiently finds the best monotonic least-squares fit to a sequence of observed data points while preserving their original order.

# Bias-corrected percentile method

Bias-corrected percentile method is an enhanced bootstrap technique for constructing confidence intervals that adjusts for both bias and skewness in the sampling distribution, offering improved accuracy over the standard percentile method. In dose-finding studies, isotonic regression typically derives point estimates (e.g., ED90) via linear interpolation, while the bias-corrected percentile method computes interval estimates; bootstrap resampling implement the robustness of interval estimates.

# Centered isotonic regression

Centered isotonic regression is a modified isotonic regression that estimates a monotonic function centered around a target parameter, minimizing the sum of squared errors in transformed space. In dose-finding studies, the non-decreasing property of standard isotonic regression can create mathematical challenges when inverting the function to derive dose estimates. Centered isotonic regression addresses this limitation by generating a strictly increasing function, which better aligns with the biologically imperative monotonicity of drug response cumulative distribution functions. This approach simultaneously circumvents mathematical complexities inherent in dose inversion procedures.

# Repeated measures analysis of variance

Repeated Measures analysis of variance analyzes within-subject changes across ≥ 3 timepoints or conditions while accounting for intra-individual correlations. Critical assumptions: ***A.*** Normality: Residuals at each timepoint should be normally distributed. ***B.*** Sphericity: Variances of differences between all pairs of repeated measurements must be equal. ***C.*** Tested via Mauchly's test. ***D.*** Homoscedasticity: Equal error variances across experimental groups. ***E***. Other assumptions.

# Generalized estimating equation

Generalized estimating equation is a semi-parametric method for analyzing correlated data (e.g., longitudinal/repeated measures). This model demonstrates considerable robustness in statistical inference.
