# Supplementary material for: Commentary: Determination of the 95% effective dose of remimazolam tosylate in anesthesia induction inhibits endotracheal intubation response in senile patients
Source: Front Pharmacol. 2025 Sep 2;16:1555758. doi: 10.3389/fphar.2025.1555758 (PMC12436137; doi:10.3389/fphar.2025.1555758)
Supplement: Supplementary file 2 [file DataSheet1.docx]

# Appendix I R script of the μ3 estimator and 95% CI

1. options(digits = 10)

2. data0<-read.csv("name.csv",header = T)

3. IR<-function(data0){

4. freq0<-as.data.frame(table(data0[,1]))

5. positive<-c(1:nrow(freq0))

6. p<-c(1:nrow(freq0))

7. freq1<-cbind(freq0,positive,p)

8.

9. for (i in 1:nrow(freq1)) {

10. freq1$positive[i]<-sum(data0[,1]==freq1$Var1[i]&data0[,2]==1)

11. }

12. freq1$p<-freq1$positive/freq1$Freq

13. c0<-as.numeric(as.character(freq1$Var1))

14. p0<-as.numeric(as.character(freq1$p))

15. library(isotone)

16.

17. p1<-gpava(y=p0,z=c0)

18. }

19.

20. p1<-IR(data0)

21. c0<-unlist(p1[2])

22. p0<-unlist(p1[3])

23. ap<-unlist(p1[1])

24.

25. finddose<-function(Γ,z,x){

26.

27. if(Γ<min(x)){

28.

29. if(x[2] == x[1]){ μ3<-Γ/x[1]*z[1] }else{

30. μ<-(Γ-x[1])/(x[2]-x[1])*(z[2]-z[1])+z[1]}

31.

32. }else if(Γ>max(x)){

33. km<-length(x)

34. p_max<-x[km]-x[km-1]

35. if(p_max==0){μ<-Γ/x[km]*z[km]}else{

36. μ<-(Γ-x[km-1])/p_max*(z[km]-z[km-1])+z[km-1]}

37.

38. }else if(Γ %in% x){

39. sameX<- match(Γ,x)

40. μ<-mean(z[sameX])

41.

42. }else{

43. k<-findInterval(Γ,x)

44. dp<-x[k+1]-x[k]

45. if(dp==0){μ3<-0.5*(z[k]+z[k+1])}else{

46. μ<-(Γ-x[k])/dp*(z[k+1]-z[k])+z[k]}

47. }}

48.

49. u3<-finddose(0.95,c0,ap)

50. names(u3)<-"thet"

51.

52. boots<-function(Γ,data){

53. df0<-c(1:2000)

54. for(i in 1:2000){

55.

56. dataS<-function(data){

57. rowS<- sample(1:nrow(data),nrow(data),replace = TRUE)

58. data[rowS,]}

59.

60. datai<-dataS(data)

61. p1_i<-IR(datai)

62. df0[i]<-finddose(Γ,unlist(p1_i[2]),unlist(p1_i[1]))

63. }

64.

65. mean(df0)

66. }

67.

68. u_mean<-boots(0.95,data0)

69.

70. findp<-function(dose,z,x){

71. h<-findInterval(dose,z)

72. if(x[h+1]==x[h]){p<-x[h]}else{

73. p<-(dose-z[h])/(z[h+1]-z[h])*(x[h+1]-x[h])+x[h]}

74. }

75.

76. p_mean<-findp(u_mean,c0,ap)

77. names(p_mean)<-"Probability"

78.

79. doseCI<-function(p){

80. z0<-qnorm(p)

81. za<-qnorm(0.975)

82. a1<-pnorm(2*z0-za)

83. a2<-pnorm(2*z0+za)

84. u1<-finddose(a1,c0,ap)

85. u2<-finddose(a2,c0,ap)

86. c(u1,u2)}

87.

88. CI_95<-doseCI(p_mean)

89. names(CI_95)<-c("CI_L","CI_U")

90.

91. list(data0,c0,p0,ap,u3,CI_95)
